# A comparison of bacterial colonization between nasogastric and orogastric enteral feeding tubes in infants in the neonatal intensive care unit

**DOI:** 10.1038/s41372-022-01452-z

**Published:** 2022-07-15

**Authors:** Kannikar Vongbhavit, Lauren K. Salinero, Karen M. Kalanetra, Chad Masarweh, Alice Yu, Diana H. Taft, David A. Mills, Mark A. Underwood

**Affiliations:** 1 Department of Pediatrics, HRH Princess Maha Chakri Sirindhorn Medical Center, Srinakharinwirot University, Nakornayok, Thailand; 2 Division of Neonatology, Department of Pediatrics, University of California Davis, Sacramento, CA USA; 3 Department of Food Science and Technology, University of California Davis, Davis, CA USA

**Keywords:** Predictive markers, Metabolism

## Abstract

**Objective:**

Feeding tubes harbor microbial contaminants; studies to date have not explored differences between orogastric (OG) and nasogastric (NG) tube biofilms. We sought to extend a previous analysis by comparing bacterial colonization by location (OG v NG) and by evaluating clinical factors that may affect tube bacterial populations.

**Study design:**

The pharyngeal segments of 41 infant feeding tubes (14 OG and 27 NG) from 41 infants were analyzed by next generation 16 S rRNA sequencing on the MiSeq platform.

**Results:**

At the phylum level, Proteobacteria had the highest relative abundance of both OG and NG tubes. At the genus/species level, nine taxa differed significantly between OG and NG tubes. Alpha and beta diversity analyses showed significant differences between OG and NG tubes with relatively little contribution from clinical factors.

**Conclusion:**

The route of feeding tube insertion (oral vs nasal) had a greater impact on bacterial colonization than the assessed clinical factors.

## Introduction

Enteral feeding tubes are commonly used in the Neonatal Intensive Care Unit (NICU) due to immaturity of coordination of sucking, swallowing and breathing or to respiratory, cardiovascular, gastrointestinal, or neurologic disease. However, enteral feeding systems rapidly develop microbial biofilms [1–3]. Feeding tubes may therefore serve as reservoirs for pathogens causing infection in the infant or transmission between patients by healthcare providers [4, 5], as demonstrated by shared bacterial strains between infants in the NICU [4, 6], This may be of particular concern as many of the strains isolated from feeding tubes are resistant to multiple antibiotics [3]. Many of the species of bacteria found in feeding tubes are nosocomial pathogens including members of the family Enterobacteriaceae*, Enterococcus* spp. and coagulase-negative *Staphylococcus* spp. [1, 3]

Feeding tubes are inserted by the nasogastric (NG) route or by the orogastric (OG) route. The nasopharynx (the upper region of the throat behind the nose) is lined by ciliated and columnar epithelium, while the oropharynx, located immediately behind the mouth, is lined by a nonkeratinized stratified squamous epithelium. The nasopharynx and the oropharynx become rapidly colonized at birth with a highly diverse ‘pioneer’ microbiome, with significant differences appearing between adjacent anatomical niches within the first few days of life [7, 8]. The nostrils harbor bacteria from the genera *Corynebacterium* and *Staphylococcus* [9–11], while the oropharynx harbors species from the genera *Streptococcus*, *Haemophilus*, *Neisseria*, and to a lesser extent *Staphylococcus* and various anaerobic bacteria [9, 12]. Maternal microbiota account at least in part for infant microbiome profiles, and there are associations between infant upper respiratory tract microbial evolution and external factors (e.g. vaginal versus cesarean delivery, breast versus formula feeding, and antibiotic exposure) [7, 13]. Discovery of the short and long-term clinical implications of colonization of the neonatal intestinal tract is still in its infancy. A better understanding of both the composition and function of colonizing microbes is needed to assess the influence of perturbations in this community on the developing immune system.

This study aimed to refine understanding of differences in bacterial colonization between nasogastric and orogastric enteral feeding tubes in hospitalized infants. We used 16 S rRNA gene sequencing technologies to characterize the bacterial composition in feeding tubes removed from NICU patients. In addition, clinical factors such as mode of delivery, gestational age, type of feeding, and antibiotic administration that influence biofilm formation were analyzed. We have previously demonstrated antimicrobial resistance genes in the pharyngeal, esophageal and gastric sections of infant feeding tubes [14]. In this study, we focused on the pharyngeal segments of the feeding tubes to compare colonization based on tube insertion site (nasal vs oral) and to explore how clinical factors influence colonization.

## Methods

### Sample collection

Feeding tubes were collected at the University of California Davis Medical Center NICU for 4 months (August to December 2015) as previously described [14]. All protocols were approved by the University of California Davis Institutional Review Board (reference number 753294-4), informed consent was obtained from the parents or legal guardians of all study enrollees and the study was performed in accordance with the Declaration of Helsinki. Participation in this study did not influence patient care. All infants in the NICU with a feeding tube in place during the sample collection period were eligible for enrollment in the study. None of the infants were treated with probiotics.

Following their scheduled removal by NICU personnel, feeding tubes were placed in sterile sleeves and frozen at −40 °C. The type of nutrition given to each subject was recorded on a weekly basis during their participation in the study. Additionally, the enrollee’s electronic medical records were examined to document feeding tube dwell time, route of feeding tube placement, gestational age of the patient, use of acid suppressors or antibiotics, and diagnoses of necrotizing enterocolitis (NEC), sepsis, and spontaneous intestinal perforation (SIP).

Ninety-seven feeding tubes were collected from 47 infants in the original study with each tube separated into pharyngeal, esophageal, and gastric segments. In the current study, we analyzed only the data from the pharyngeal segments as these were most likely to be influenced by insertion site (oral vs nasal).

### Sample preparation

Each tube was flushed with 1 mL sterile PBS followed by 0.5 mL of air. The pharyngeal segment–the 3 cm section beginning 5 cm below the point where the tube exited the body (tube insertion depth)–was split lengthwise and cut into approximately 2 mm pieces with a sterile scalpel.

### DNA library construction and sequencing

DNA was extracted from the prepared tube and analyzed as previously described [14]. Briefly, the V4 region of the 16 S rRNA gene was amplified in triplicate with barcoded primers from each sample. Successful amplification was confirmed via gel electrophoresis, and then samples were consolidated and purified using the Qiagen QIAquick PCR Purification Kit (Qiagen, Hilden, Germany). Purified barcoded amplicons were then submitted to the UC Davis Genome Center DNA Technologies Sequencing Core for paired-end library preparation and sequencing using the Illumina MiSeq DNA sequencing system (Illumina, San Diego, CA, USA).

Resulting reads were demultiplexed using Sabre [15], then imported into QIIME2–2018.4–2018.4 [16]. Bases before bp 22 and after bp 210 were trimmed from the forward read. Bases before bp 24 and after bp 210 were trimmed from the reverse read. The trimmed reads were then processed using DADA2 [17]. The resulting replicon sequence variant (RSV) table, phylogenetic tree, representative sequences, and taxonomic assignments were exported from QIIME2–2018.4 [18] for use in downstream analysis in R 3.4.3 statistical software [19].

Pharyngeal segments with a sequencing depth of fewer than 1500 reads were excluded after excluding RSVs detected in the kit control samples. Samples were rarefied to 1528 reads, which was the read depth of the lowest included sample after excluding kit contamination associated RSVs, leaving 73 pharyngeal segments from 41 infants. We selected one pharyngeal segment (NG or OG) from each infant for more detailed analysis, selecting tubes collected from similar periods and dwell times when possible (27 infants with an NG tube and 14 infants with an OG tube).

### Statistical analysis

Statistical analysis was completed in R version 3.4.3 statistical software and QIIME2–2018.4. For the 16 S rRNA gene sequencing analysis, the sequencing depth of samples in the RSV table was checked, and all samples within five times the sequencing depth of the highest negative control were excluded. Sequences were then rarefied to the read depth of the sample with the fewest reads using the vegan package 2.4–2 [20, 21]. Alpha diversity was analyzed using the Shannon and Simpson indices. Differences in these pharyngeal samples between NG tubes and OG tubes were tested using a Wilcoxon rank-sum test.

Beta-diversity was analyzed separately by the Bray–Curtis, Jaccard, and Unweighted UniFrac and Weighted UniFrac distances. Differences in microbial community beta diversity were explored visually using principal coordinates analysis (PCoA). Differences in beta diversity were tested using PERMANOVA as implemented by the Adonis command in vegan [22].

Analysis of the composition of microbiomes with bias correction (ANCOM-BC) [23] was used to detect differences in microbial compositions between OG and NG tubes. ANCOM-BC employs the Wilcoxon rank-sum test for identifying taxa that are differentially abundant and includes multiple hypothesis correction by the Holm-Bonferroni method; it is a valuable tool for comparing relative abundance between groups due to its capacity to control the false discovery rate at nominal levels while maintaining power. An ANCOM-BC detection *q* value <0.05 was considered significant (q values are the p values adjusted for the optimized false discovery rate). Differences in bacterial genus between OG and NG tubes and by clinical variables were determined. Clinical data analyzed included gestational age (term infant ≥37 weeks or preterm infant <37 weeks), mode of delivery (Cesarean section or vaginal delivery), milk type (breast milk, infant formula or mixed diet), and antibiotic administration.

## Results

Table 1 summarizes the demographic and clinical data of the infants in each group. There was a larger percentage of preterm infants in the OG group than the NG group (93% vs. 67%). Consequently, infants with OG tubes received intrapartum antibiotics, antenatal steroids and antibiotics on the day of birth more frequently compared to the infants with NG tubes (*p* values 0.04, 0.008, and 0.005 respectively), and the postmenstrual age at the time of feeding tube removal was lower in the OG group than the NG group. There was no significant difference in received antibiotics on the day of tube collection or the other clinical factors between infants with OG tubes and infants with NG tubes.

**Table 1:** Demographics and clinical outcomes of the cohort.

Demographic data	Infants with OG tubes (*n* = 14)	Infants with NG tubes (*n* = 27)	*p* value
Preterm (Gestational age <37 weeks, %)	13 (92.9)	18 (66.7)	0.06
Male sex (%)	6 (42.9)	16 (59.3)	0.32
Cesarean section (%)	10 (71.4)	20 (74.1)	0.86
Exclusive breast milk feeding (%)	9 (64.3)	21 (77.8)	0.36
Rupture of membranes >18 h (%)	3 (21.4)	3 (11.1)	0.37
Intrapartum antibiotic prophylaxis (%)	7 (50.0)	5 (18.5)	0.04
One or more dose(s) of antenatal steroids (%)	9 (64.3)	6 (22.2)	0.008
One or more dose(s) of acid suppressing agent (%)	1 (7.1)	1 (3.7)	1
Antibiotics on the day of birth (%)	14 (100)	16 (59.3)	0.005
Antibiotics on the day of tube collection (%)	7 (50)	10 (37.0)	0.42
Culture positive sepsis (%)	4 (28.6)	3 (11.1)	0.20
Necrotizing enterocolitis stage 2 or 3 (%)	1 (7.1)	1 (3.7)	1
Spontaneous intestinal perforation (%)	1 (7.1)	1 (3.7)	1
Duration of feeding tube, days (inter-quartile range)	6 (4–14)	7 (4–12)	0.71
Median gestational age at birth in completed weeks (inter-quartile range)	27.5 (25–31.5)	34 (32–38)	<0.001
Median postmenstrual age at the time of tube collection in completed weeks (inter-quartile range)	31 (28–33)	35 (34–39)	<0.001
Postmenstrual age <37 weeks at the time of tube collection in completed weeks (%)	12 (85.7)	14 (51.9)	0.03

### Bacterial abundance

**Fig. 1:**
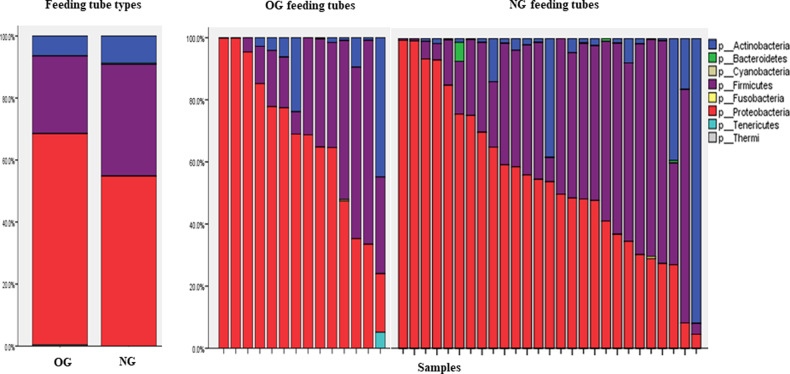
The relative abundance of bacterial phyla in the NG and OG tubes. The panel on the left shows the relative abundance of phyla in all combined OG and NG tubes. The panel on the right shows the relative abundance of each individual tube.

The most abundant phyla identified in the samples were Proteobacteria, Firmicutes, and Actinobacteria. Bacteroidetes, Cyanobacteria, Fusobacteria, Tenericutes, and Thermi were also detected. Figure 1 shows bacterial phyla in OG and NG tubes. Mean relative abundance in NG and OG tubes was Proteobacteria (49% and 59% respectively), Firmicutes (32%, and 22% respectively), and Actinobacteria (8% and 6% respectively).

There was an inverse correlation in all feeding tubes in the relative abundance of Proteobacteria and Firmicutes (Spearman correlation coefficient −0.75, *p* < 0.001*)* and Proteobacteria and Actinobacteria (Spearman correlation coefficient −0.48, *p* = 0.002). This pattern held true within the NG and OG subgroups respectively, with an inverse correlation in both the relative abundance of Proteobacteria and Actinobacteria (Spearman correlation coefficient −0.48, *p* = 0.012) and Proteobacteria and Firmicutes (Spearman correlation coefficient −0.67, *p* < 0.001) in NG tubes and in the relative abundance of Proteobacteria and Firmicutes (Spearman correlation coefficient −0.85, *p* < 0.001) in OG tubes.

A total of 15 bacterial families were detected with mean relative abundance greater than one percent (Supplementary Table 1). Enterobacteriaceae were most abundant in both feeding tubes (27% in NG tubes and 38% in OG tubes). Moraxellaceae (14%) and Staphylococcaceae (11%) were more abundant in NG tubes than OG tubes, while Streptococcaceae (10%) and Neisseriaceae (6%) were more abundant in OG tubes.

Using ANCOM-BC analysis, nine genera/species were differently expressed between NG and OG tubes (Table 2). *Corynebacterium kroppenstedtii, Streptococcus luteciae*, and *Pseudomonas* were more abundant in OG tubes than NG tubes. In contrast, *Enterococcus, Staphylococcus, Streptococcus, Neisseria, Haemophilus parainfluenzae*, and *Pseudomonas fragi*, were more abundant in NG tubes. *Pseudomonas* were more abundant in infants receiving antibiotic treatment on the first day of life (*q* < 0.01). There were no significant differences at the genus level between preterm and term infants, mode of delivery, duration of feeding tubes, and milk type feeding.

**Table 2:** Taxa with statistically significant differences in abundance between NG and OG tubes (ANCOM-BC analysis).

Phylum	Family	Genus/Species	*q*
Actinobacteria	Corynebacteriaceae	*Corynebacterium; s__kroppenstedtii*	<0.01
Firmicutes	Enterococcaceae	*Enterococcus; s__*	<0.01
	Staphylococcaceae	*Staphylococcus*	<0.01
	Streptococcaceae	*Streptococcus; s__*	<0.01
	Streptococcaceae	*Streptococcus; s__luteciae*	<0.01
Proteobacteria	Neisseriaceae	*Neisseria*	<0.01
	Pasteurellaceae	*Haemophilus; s__parainfluenzae*	<0.01
	Pseudomonadaceae	*Pseudomonas; s__*	<0.01
	Pseudomonadaceae	*Pseudomonas; s__fragi*	<0.01

### Alpha and Beta diversity analyses

**Fig. 2:**
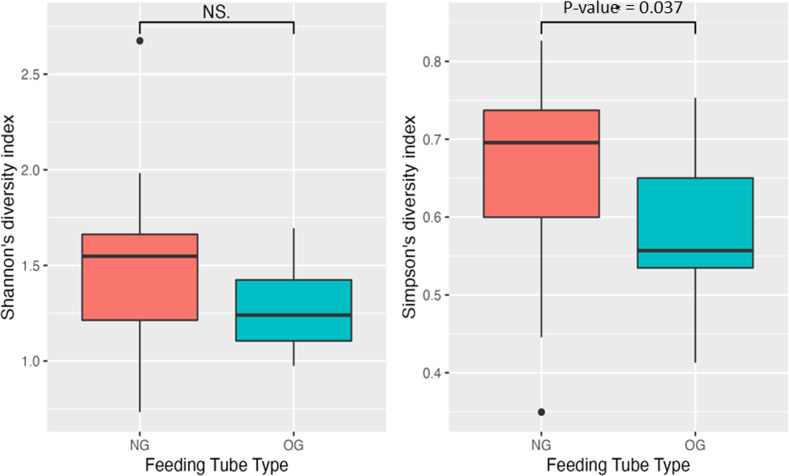
Alpha diversity as measured by Shannon’s diversity index and Simpson’s diversity index. The NG tubes had significantly higher alpha diversity than the OG tubes by Simpson’s index but not by Shannon’s index.

Alpha diversity was measured by Shannon’s diversity index and Simpson’s diversity index. The NG tubes had significantly higher alpha diversity than the OG tubes by Simpson’s index but not by Shannon’s index (Fig. 2). There were no significant differences in alpha diversity in all feeding tubes or in the subsets (NG vs OG) based on the clinical variables such as preterm/term infants, mode of delivery, dwell time of feeding tubes, antibiotics, milk type feeding, or history of NEC or SIP (Supplementary Table 2).

**Fig. 3:**
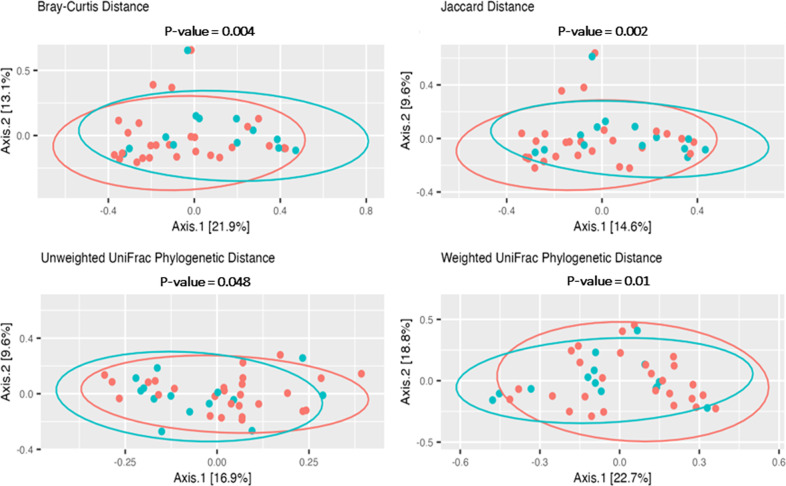
Principal-coordinate analysis (PCoA) 2D plots of beta diversity analysis between OG and NG tubes samples analyzed. Orange dots denote NG tube samples. Green dots denote OG tubes samples.

Beta diversity was measured using Bray–Curtis, Jaccard, Unweighted UniFrac, and Weighted UniFrac dissimilarity between OG and NG tubes. PERMANOVA testing for statistical significance was assessed. Microbial communities between samples collected from NG and OG tubes were significantly different based on the insertion route of the feeding tube (NG vs OG) according to Bray–Curtis dissimilarity (*p* = 0.004). Similar results were obtained using Jaccard, Unweighted UniFrac, and Weighted UniFrac (0.002, 0.048, and 0.02 respectively). This suggests the NG and OG tubes communities were significantly different. Figure 3 shows principal coordinates analysis plots (PCoA) of Bray–Curtis, Jaccard, Unweighted UniFrac, and Weighted UniFrac distances for all feeding tube samples analyzed. Supplementary Table 3 summarizes beta diversity between NG and OG and clinical criteria with all feeding tubes. Notably, antibiotic administration and duration of feeding tube placement influenced some measures of beta diversity.

## Discussion

The newborn infant microbiota is highly dynamic and undergoes rapid changes in composition through the first years of life with distinct microbial communities (both taxonomically and functionally) at specific body sites [24, 25]. Biofilms on feeding tubes are likely influenced by both bacteria from the site of placement (oral or nasal) and bacteria from the stomach and intestine. Infants often have disturbed motility manifesting as gastroesophageal reflux (often into both the nose and mouth), abdominal distention and bacterial overgrowth. Secondary effects from bacterial metabolic products or host mucosal immune responses common to both the gut and respiratory tract also likely impact these biofilms [10, 26–28]. The gut and respiratory tracts share the same embryonic origin, with mucosal surfaces composed of columnar epithelial cells that sense commensal bacteria and in turn shape local and systemic immunity as infants mature and as a function of postmenstrual age [26, 27, 29]. Feeding tube biofilms are also likely influenced by diet, antibiotics, therapeutics, and environmental exposures in the NICU [30–32]. The effect of these changes can be illustrated by antibiotic-induced alterations of neonatal gut microbiota during the crucial early postnatal period of immune competence [31–33].

In our initial study we found that biofilms on feeding tubes were dominated by *Proteobacteria* and *Firmicutes* at the phylum level with *Actinobacteria* present in smaller amounts. These bacteria have been previously reported as the most abundant components of bacterial populations in NICU patient feeding tubes, enteral feeding system extension tubing, and neonates’ gastroesophageal microbiota [1–3, 6, 34, 35]. We also found that feeding tube biofilms were influenced by infant gut microbes and that antibiotic resistance genes are common in microbes in feeding tube biofilms.

In the current study, we focused on differences between biofilms on NG and OG tubes and on correlations between clinical factors and feeding tube biofilms. We focused just on the pharyngeal sections of the feeding tubes as these are most likely to be influenced by site of insertion. We found significant differences between NG and OG tubes in relative abundance of several taxa at the family and genus/species level as well as in alpha and beta diversities. It is likely that some of the difference between NG and OG tube biofilms is related to differences in gestational age at birth (including the impact of antenatal steroids, intrapartum and postpartum antibiotics, and the need for nasal CPAP which may limit the use of NG tubes); while our analysis did not find statistical significance (*p* values of 0.07 and 0.09 in supplementary Table 3), it is certainly possible that evaluation of a larger sample size would demonstrate a significant effect of gestational age. A recent study demonstrated a lower incidence of aspiration and tube displacement in NG tubes compared to OG tubes, and that infants regained birth weight more quickly with NG feeding than with OG tube feeding [36]. Our data raise the question of whether differential colonization of the feeding tube is one of the mechanisms underlying these differences.

The predominance in both OG and NG tubes of Proteobacteria at the phylum level and Enterobacteriaceae at the family level has clinical relevance as these organisms have been described as a marker of intestinal dysbiosis [37] and associated with increased risk of NEC in preterm infants [38]. It is noteworthy that there were no significant differences between NG and OG tubes at the genus/species level for the Enterobacteriaceae most commonly associated with NEC and late onset sepsis (*Escherichia* and *Klebsiella*). We found differences between OG and NG tubes at the genus/species level for *Pseudomonas*, with differing species more common in each. If this is confirmed in future studies, understanding the mechanisms underlying this differential preference would be valuable, as many *Pseudomonas* species are opportunistic pathogens, forming penicillin- and other beta-lactam-resistant biofilms together with other species colonizing hospitals and causing NICU outbreaks [39, 40].

Our finding of increased Moraxellaceae and Staphylococcaceae in NG tubes and Streptococcaceae and Neisseriaceae in OG tubes is consistent with differences in colonization of the nasopharynx and oropharynx. Future studies of the impact of altering the nasal microbiota (e.g. with decolonization of methicillin resistant strains of *S. aureus*) on feeding tube biofilms or comparisons of biofilms on NG tubes between infants colonized with coagulase positive and negative staphylococci would be valuable.

Previous studies showed evidence for the presence of some infant gut-associated strains in the NICU room environment and for exchange of those strains between infant and room environments [41]. Future studies investigating the impact of NICU environmental cleaning regimens on feeding tube biofilms and outbreaks of disease from common organisms in these biofilms are needed. None of the infants in this study received probiotic dietary supplements; it would be valuable to explore the influence of differing probiotic strains on feeding tube colonization. There may also be value in exploring the clinical impact of feeding tubes coated with commensal organisms or antimicrobial molecules.

One drawback to the analytical techniques used in this study is that they do not distinguish pathogens from commensal strains. This has clinical relevance, as both a lack of commensal bacteria and an overabundance of potentially pathogenic bacteria have been associated with life-threatening diseases [42]. Additionally, many strains of *Enterobacteriaceae* isolated from NICU feeding tubes have been found to be resistant to antibiotics [3]. While numerous microbial communities within individual body sites have been described [24, 43–46], associations between the microbiota across multiple body sites or systems are less well studied [47, 48].

This study has several additional limitations. Cohort studies are helpful to establish associations but not causality. The sample size is small and was based on feasibility rather than a sample size calculation. In addition, selecting a single tube for those infants with several tubes collected for this comparison raises the possibility of increased confounding by clinical factors. This study also only examined bacteria. Future studies of the potential roles for viruses, fungi and archaea in feeding tube biofilms, and the impact of human milk components (e.g. immunoglobulins, lactoferrin, lysozyme and human milk oligosaccharides) on feeding tube microbial communities may have value.

## Conclusion

Neonatal feeding tubes are dominated by Proteobacteria, particularly Enterobacteriaceae, Moraxellaceae and Neissieraceae, and Firmicutes, particularly Staphylococcaceae and Streptococceae. Community composition is dissimilar between feeding tubes placed through the nasal vs oral route with increased Moraxellaceae and Staphylococcaceae in the NG tubes and Neisseriaceae and Streptococcaceae in the OG tubes. The impact of differences in gestational age, delivery type, feeding type and other clinical factors appeared to be less important than site of insertion in this small cohort. Further studies to confirm differences between OG and NG tubes and the potential clinical impact of site of insertion are indicated.

## Supplementary information

Supplemental Table 1

Supplemental Table 2

Supplemental Table 3

## Data Availability

Data available on request from the authors.
