# Supplementary material for: A comparison of bacterial colonization between nasogastric and orogastric enteral feeding tubes in infants in the neonatal intensive care unit
Source: J Perinatol. 2022 Jul 15;42(11):1446–52. doi: 10.1038/s41372-022-01452-z (PMC9616717; doi:10.1038/s41372-022-01452-z)
Supplement: Supplementary file 1 — Supplemental Table 1 [file 41372_2022_1452_MOESM1_ESM.docx]

**Supplementary Table 1:** Bacterial families between OG and NG tubes with mean relative abundance >1%

|  | Mean relative abundance | |
| --- | --- | --- |
| Family | NG tubes | OG tubes |
| Enterobacteriaceae | 27% | 38% |
| Moraxellaceae | 14% | 8% |
| Staphylococcaceae | 11% | 3% |
| Streptococcaceae | 8% | 10% |
| Pasteurellaceae | 4% | 2% |
| Micrococcaceae | 4% | 2% |
| Corynebacteriaceae | 3% | 4% |
| Enterococcaceae | 3% | 1% |
| Gemellaceae | 3% | 1% |
| Aerococcaceae | 2% | 0% |
| Xanthomonadaceae | 2% | 0% |
| Pseudomonadaceae | 1% | 4% |
| Planococcaceae | 1% | 1% |
| Lactobacillaceae | 1% | 4% |
| Neisseriaceae | 0% | 6% |
