# Supplementary material for: A comparison of bacterial colonization between nasogastric and orogastric enteral feeding tubes in infants in the neonatal intensive care unit
Source: J Perinatol. 2022 Jul 15;42(11):1446–52. doi: 10.1038/s41372-022-01452-z (PMC9616717; doi:10.1038/s41372-022-01452-z)
Supplement: Supplementary file 2 — Supplemental Table 2 [file 41372_2022_1452_MOESM2_ESM.docx]

**Supplementary Table 2:** Alpha diversity p values based on variable criteria of all feeding tubes and between OG and NG tubes

|  | All feeding tubes | | Between OG and NG tubes | |
| --- | --- | --- | --- | --- |
| Clinical variables | Shannon | Simpson | Shannon | Simpson |
| NG tubes vs OG tubes | 0.05 | 0.037 | NA | NA |
| Preterm vs Term infants | 0.69 | 0.41 | 0.28 | 0.17 |
| Postmenstrual age at the time of tube collection in completed weeks  (<37 weeks vs >37 weeks) | 0.69 | 0.41 | 0.28 | 0.18 |
| Vaginal delivery vs Cesarean section | 0.38 | 0.75 | 0.18 | 0.18 |
| Initial antibiotic administration: yes vs no | 0.64 | 0.66 | 0.14 | 0.10 |
| Breast milk vs  Infant formula or Mixed type | 0.92 | 1 | 0.21 | 0.20 |
| Duration of feeding tube in place  (< 7 days vs >7 days) | 0.27 | 0.51 | 0.21 | 0.17 |
