# Supplementary material for: A comparison of bacterial colonization between nasogastric and orogastric enteral feeding tubes in infants in the neonatal intensive care unit
Source: J Perinatol. 2022 Jul 15;42(11):1446–52. doi: 10.1038/s41372-022-01452-z (PMC9616717; doi:10.1038/s41372-022-01452-z)
Supplement: Supplementary file 3 — Supplemental Table 3 [file 41372_2022_1452_MOESM3_ESM.docx]

**Supplementary Table 3:** Beta diversity between OG and NG tubes and based on variable criteria of all feeding tubes

|  | Bray-Curtis | Jaccard | Unweighted Unifrac | Weighted Unifrac |
| --- | --- | --- | --- | --- |
| Clinical variables | p-value | p-value | p-value | p-value |
| NG tubes vs OG tubes | 0.004 | 0.002 | 0.048 | 0.02 |
| Preterm vs Term infants | 0.09 | 0.07 | 0.19 | 0.15 |
| Postmenstrual age at the time of tube collection in completed weeks  (<37 weeks vs >37 weeks) | 0.09 | 0.08 | 0.19 | 0.14 |
| Vaginal delivery vs Cesarean section | 0.23 | 0.25 | 0.48 | 0.42 |
| Antibiotic administration on the day of birth: yes vs no | 0.03 | 0.04 | 0.02 | 0.09 |
| Breast milk vs Infant formula or Mixed type | 0.75 | 0.77 | 0.89 | 0.93 |
| Duration of feeding tube in place  (< 7 days vs >7 days) | 0.07 | 0.03 | 0.01 | 0.06 |
